# The Feasibility and Positive Effects of Wuqinxi Exercise on the Cognitive and Motor Functions of Patients with Parkinson's Disease: A Pilot Study

**DOI:** 10.1155/2021/8833736

**Published:** 2021-03-29

**Authors:** Mengyue Shen, Yan-Ling Pi, Zhenlan Li, Tao Song, Kuncheng Jie, Tian Wang, Wei Guo, Jie Zhuang, Zhen Wang

**Affiliations:** ^1^School of Martial Arts, Shanghai University of Sport, Shanghai, China; ^2^Shanghai Punan Hosptial of Pudong New District, Shanghai, China; ^3^School of Sport Science, Shanghai University of Sport, Shanghai, China

## Abstract

**Introduction:**

Parkinson's disease (PD) is a chronic degenerative disease of the central nervous system common in middle-aged and elderly people, which has a serious impact on patients' cognitive and motor functions. Exercise can improve the nonmotor symptoms of PD patients, but the optimal type of exercise for the cognitive function of patients is unclear. Therefore, the purpose of this study is the impact of 12 weeks of Wuqinxi exercise on the cognitive and motor function in PD patients.

**Methods:**

Thirty PD patients participated in the study and were randomly assigned to two groups: Wuqinxi group (*n* = 15) or stretching group (*n* = 15). All the participants performed a 12-week exercise program twice a week, 90 min/session. The assessments were conducted before and after exercise intervention, included cognitive function (frontal assessment battery (FAB); Stroop test I and II), motor functions (Unified Parkinson's Disease Rating Scale Part III (UPDRS-III); timed up and go (TUG)).

**Results:**

We found the FAB and Stroop I scores were significantly higher in the Wuqinxi group than in the stretching group. Participants in the Wuqinxi group significantly improved their UPDRS-III (17.73 ± 9.88) and TUG (10.50 ± 1.79) score after 12 weeks of training intervention.

**Conclusion:**

The results show that the use of Wuqinxi for rehabilitation therapy for cognition is feasible, widely accepted, and effective in patients with Parkinson's disease. This study provides preliminary evidence for further large-scale and controlled studies.

## 1. Introduction

Parkinson's disease (PD) is a progressive neurodegenerative disease characterized by dopamine (DA) depletion [1]. The patient has characteristic motor symptoms and nonmotor symptoms such as sleep disorders, cognitive impairment, anxiety, and depression [2]. Among them, cognitive dysfunction aggravates motor symptoms. According to reports, the incidence of dementia in PD patients is about 12%–30%, which brings a huge burden to patients' lives and families [3]. Dementia is characterized by the progressive cognitive decline associated with behavior disturbances and differential in performing the activities of daily living [4]. Another common factor in this neuropathology is the impairment of executive functions [5, 6]. Notably, executive deficits can occur at any stage of the disease and can be progressive [7]. However, the question of impairment of executive function remains unresolved.

Exercise can promote the growth of brain cells and the diversity and complexity of neuronal communication and help improve the executive function of the elderly [8]. Wuqinxi exercise belongs to the healthy Qigong exercise. Previous studies have shown that Wuqinxi exercise has been used to improve osteoporosis in the elderly [9], attention concentration ability [10], immune function [11] and exercise capacity [12], and reduce cardiovascular disease and metabolic syndrome [13]. Although more and more studies have shown the great potential of Wuqinxi, there is no research to explore the impact of Wuqinxi on the executive function of PD patients.

At present, many methods of testing executive function have been developed. For example, the Stroop color and word test studies the classic paradigm of executive function, which can measure inhibition control ability [14]. We also used the frontal assessment battery (FAB) assessment tool, which has been shown to be superior to the mini-mental state examination (MMSE), as a screening tool for neurodegenerative diseases involving the frontal lobe. It is rarely used in studies of PD patients [15].

Therefore, this study aims to systematically evaluate the process and scientific feasibility of the experimental design, compare the effects of Wuqinxi exercise and stretching exercises on PD cognition and motor function, and provide a new and effective rehabilitation exercise for Parkinson's patients. We hypothesize that the 12-week Wuqinxi exercise is effective in improving PD cognitive and motor function and is better than the stretching group.

## 2. Methods

### 2.1. Participants

PD patients were recruited through Shanghai University of Sport or Xinhua Hospital Affiliated to Shanghai Jiaotong University School of Medicine by referrals from neurologists and/or physical therapists and distribution of the study information to local support groups for persons with PD. The inclusion criteria for the study participants included a clinical diagnosis of PD, ages between 55 and 80 years, and with a disease severity from mild to moderate level (rating from 1 to 3 out 5) according to the Hoehn and Yahr scale [16]; drug treatment is stable, the patient can walk independently or with the aid of walkers. The exclusion criteria included currently involved in any behavioural or pharmacological intervention study or instructor-led exercise training program; serious organic diseases (heart disease, hypertension, tuberculosis, nephritis, etc.) in the past two years; history of alcoholism, smoking, and visual or hearing impairment; a mini-mental state examination score lower than 24 [15] and deep brain stimulation surgery (DBS). All interventions are carried out during the drug “on” phase.

### 2.2. Study Design

We designed a randomized clinical trial to compare the effects of exercise at 3 months in a group of patients assigned to Wuqinxi classes with the effects in groups assigned to stretching classes. Each group participated in a 90-minute class that met twice weekly for 12 weeks. The research was supported by the Ethics Committee of Shanghai Sport University and obtained written informed consent from all participants. The research was registered in China Clinical Trial Registry (ID: ChiCTR1800016570).

### 2.3. Screening and Randomization

Research staff screened patients by telephone; those who met prescreening criteria underwent an in-person evaluation and baseline assessment. We used stratified random sampling (H&Y period). Using computer-generated random number sorting (Stata V.12.0, Statacorp, Texas, USA), a random number is placed in a sealed envelope at a ratio of 1 : 1, and the participants are drawn by random numbers from the envelope. The participants are randomly assigned to Wuqinxi group or stretching group. Outcome assessors were unaware of group assignments.

### 2.4. Exercise Interventions

#### 2.4.1. Wuqinxi Exercise

**Figure 1:**
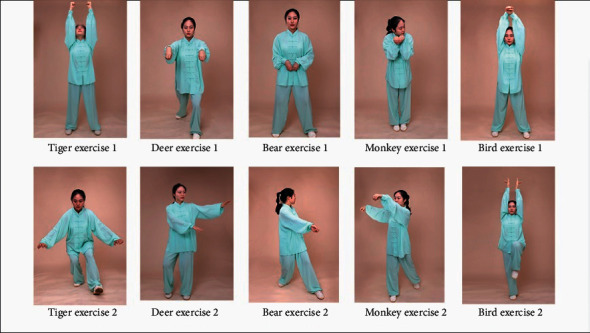
Illustrations of Wuqinxi exercise maneuvers. The agreement includes ten actions such as tiger raising, tiger seizing, deer colliding, deer running, bear swaying, bear rubbing, ape being alert, ape plunking, bird stretching, and bird flying.

The protocol consisted of ten movements (two movements in each of the five animals). During the training, it is required to imagine the movement characteristics of the five animals (tiger, deer, bear, ape, and bird), emphasizing the coordination of body movements, breathing and mind, loosening the limbs, and relaxing the spirit. A professional trainer conducts practice guidance twice a week, 90 minutes each time. After the exercise, each patient is required to fill in the exercise log, including the heart rate, rest time, and exercise time during each training period (Figure 1).

#### 2.4.2. Stretching Exercise

This control method is formulated by a physical therapist. It is a low-intensity activity stretching method that consists of sitting and standing stretching. The range of movement includes the upper body (neck, shoulders, upper back, chest, and arms) and lower extremities (quadriceps, triceps, calf muscles, and hips), with joint extension, bending, and trunk rotation suitable for the physical characteristics of the elderly. The main breathing method is abdominal breathing, and the emphasis is on coordination of inhalation and movement to relax the muscles. The stretching group is also conducted under the guidance of the coach, twice a week, 90 minutes each time, a total of 12 weeks.

### 2.5. Outcome Assessments

#### 2.5.1. Primary Outcomes

Stroop colour and word test [17] is a neuropsychological test widely used in clinical experimental study. It is the best evidence to prove that the patient's executive function is the ability to suppress interference. The ST-I is used as a measure of processing speed, and the ST-II is used as a measure of selective attention and inhibition. The frontal assessment battery (FAB) is a short bedside screening instrument that evaluates six domains of frontal lobe function, namely, conceptualization [18]. The Montreal Cognitive Assessment Scale (MoCA) is a rapid assessment scale that covers cognitive domains such as executive function, language, orientation, calculation, conceptual thinking, memory, visuospatial perception, attention, and concentration [19].

#### 2.5.2. Secondary Outcomes

UPDRS is an internationally used scale for assessing the degree of disease and motor function of Parkinson's patients. There are a total of four parts. UPDRS-III was selected for evaluation in this study [20] score ranging from 0 to 56, with lower values indicating less motor disability. The timed up and go (TUG) test is a well-known clinical test for assessing of mobility and fall risk [21]. The subject was instructed to stand up from the sitting position indicated by the examiner, walk 3 meters in a comfortable place, turn around, walk back to the chair, and sit down.

### 2.6. Statistics Analysis

Statistical data analysis was performed using IBM SPSS 25.0 (IBM Corp., Armonk, NY) software, with descriptive statistics (mean and standard deviation). The paired *t*-test was used to analyze the baseline and motor function data before and after the exercise intervention in the group. The normality of the data was tested using the Shapiro–Wilk test. The effects of the exercises on cognitive function were examined using two-way repeated-measures ANOVA, with the group (Wuqinxi or stretching) serving as the between-subject factor and time (baseline, 12 weeks) serving as the within-subject factor. Significant differences in ANOVA were analysed by the Bonferroni post hottest. *P* value <0.05 indicates significant difference; *P* value <0.01 indicates extremely significant difference.

## 3. Results

### 3.1. Baseline Characteristics of the Participants

**Figure 2:**
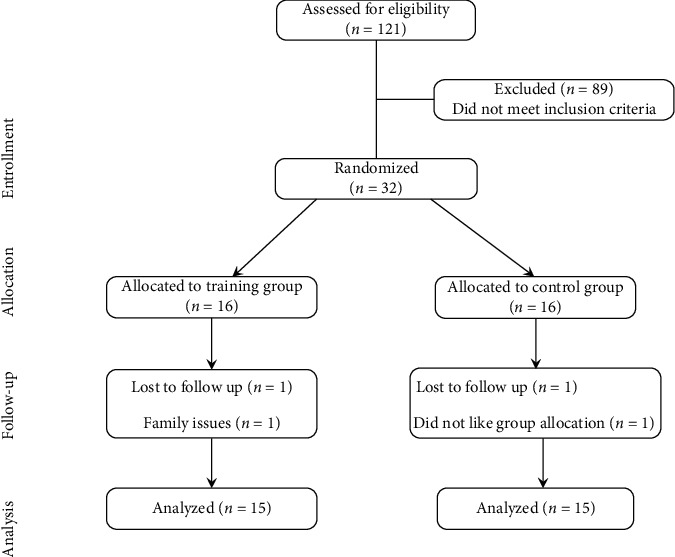
Patient flow diagram.

Of the 121 people who were screened, 32 were registered and randomized, while 89 subjects refused to participate before randomization. In the end, 30 patients were randomly assigned to the traditional Wuqinxi exercise training group (*N* = 15) or the stretching exercise training group (*N* = 15). One person in the Wuqinxi group withdrew from training because he needed to take care of his family, and one person in the stretching group withdrew because he did not like exercise (Figure 2).

Table 1 shows the demographic and clinical characteristics of the patients. There were no significant differences in demographic and baseline variables between the two groups, indicating that randomization was acceptable. During the study, there were no reports of fall in both groups.

### 3.2. Exercise Intensity

In terms of exercise intensity, the target heart rate of PD patients was calculated according to the formula HRmax = 208 − (0.7 × age), and it was maintained for about 20 minutes during training [22]. The heart rate of PD patients during training was monitored by Polar Team2 heart rate telemeter. Exercise intensity did not differ significantly between the Wuqinxi group and stretching group. Exercise intensity in the two groups was between 50% and 70%, which can be considered relatively comfortable aerobic exercise [23].

### 3.3. Outcomes

#### 3.3.1. Cognitive Ability

**Figure 3:**
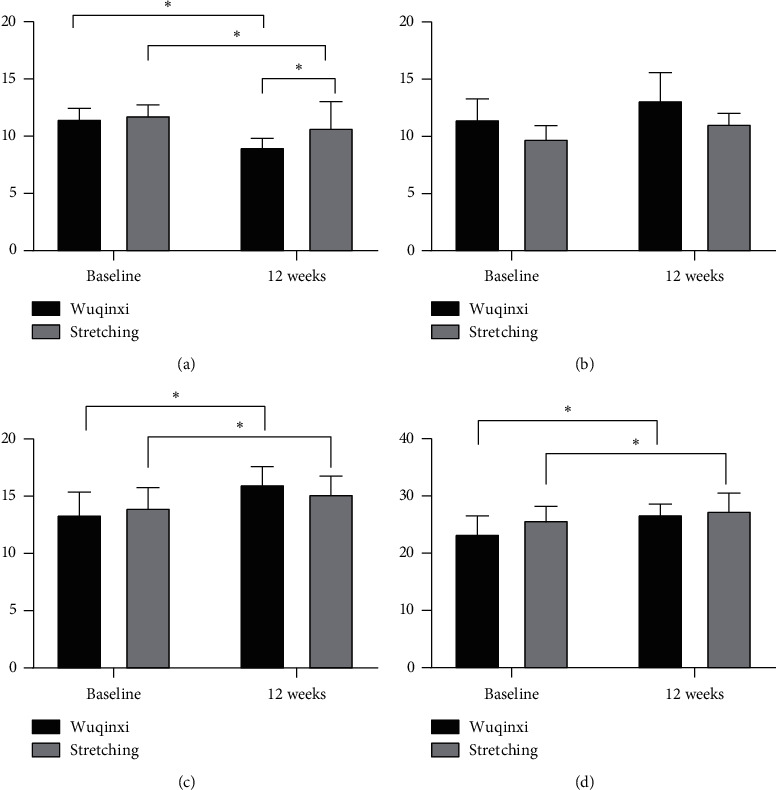
Changes in cognitive function after 12 weeks of intervention. (a) ST-I, part I of the Stroop test. (b) ST-II, part II of the Stroop test. (c) FAB, the frontal assessment battery. (d) MoCA, Montreal Cognitive Assessment. ^*∗*^*P* < 0.05 for the indicated comparisons.

For the ST-I score, after 12 weeks of intervention, there is a significant difference in time (*P* = 0.011), but the stretching group (1.69 ± 0.62) is higher than the Wuqinxi group (0.32 ± 0.33); there is a significant difference between the two groups (*P* = 0.026), the Wuqinxi group (2.47 ± 0.47) is greater than the stretching group (1.10 ± 0.47); and there is a significant interaction between time and group (*F* (1,28) = 4.229, *P* = 0.049). The Wuqinxi group is better than the stretching group (Figure 3(a)).

For the ST-II score, after 12 weeks of intervention, there is a significant difference in time (*P* = 0.004), but the Wuqinxi group (2.04 ± 0.66) is higher than the stretching group (1.68 ± 0.54); there is a significant difference between the two groups (*P* < 0.001); the Wuqinxi group (1.65 ± 0.40) is greater than the stretching group (1.30 ± 0.40); but there was no significant interaction between time and group (*F* (1,28) = 4.229, *P* = 0.545) (Figure 3(b)).

For the FAB score, after 12 weeks of intervention, there is no significant difference in time, but the stretching group (0.66 ± 0.33) is higher than the Wuqinxi group (0.60 ± 0.99); there is a significant difference between the two groups (*P* = 0.023), the Wuqinxi group (2.66 ± 0.47) is greater than the stretching group (1.13 ± 0.47), and there is a significant interaction between time and group (*F* (1,28) = 5.305, *P* = 0.029]). The Wuqinxi group is better than the stretching group (Figure 3(c)).

For the MOCA score, after 12 weeks of intervention, there is no significant difference in time, but the stretching group (2.40 ± 1.01) is higher than the Wuqinxi group (0.60 ± 0.99); there is a significant difference between the two groups (*P* = 0.001), the Wuqinxi group (3.40 ± 0.46) is greater than the stretching group (1.66 ± 0.46), and there is a significant interaction between time and group (*F* (1,28) = 7.094, *P* = 0.013). The Wuqinxi group is better than the stretching group (Figure 3(d)).

#### 3.3.2. Motor Function

**Figure 4:**
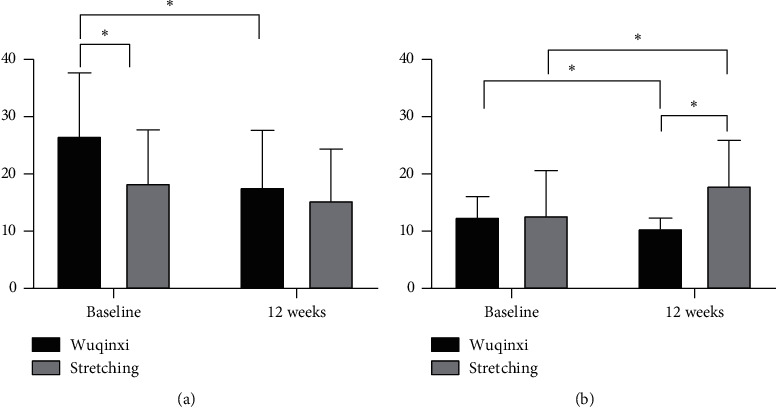
Changes in motor function after 12 weeks of intervention. (a) UPDRS-III, Unified Parkinson's Disease Rating Scale III. (b) TUG, the timed up and go. ^*∗*^*P* < 0.05 for the indicated comparisons.

After 12 weeks of intervention, the UPDRS-III score of PD patients in the Wuqinxi group decreased from 26.67 (10.99) to 17.73 (9.88) previously measured, a 33% decrease, with a statistical difference (*P* = 0.021) (Figure 4(a)). The score before and after the TUG Wuqinxi group decreased by 16% from 12.52 (3.52) to 10.50 (1.79), and the change was statistically different (*P* = 0.007); the TUG score of PD patients in the stretching group increased from 12.80 (7.77) to 17.97 (7.88), an increase of 28%, and the change before and after was statistically different (*P* = 0.004), indicating that the walking ability of the stretching group decreased compared with that before the intervention, which was statistically significant, but had no actual clinical significance (Figure 4(b)).

## 4. Discussion

Our study proved for the first time that the Wuqinxi exercise is effective in the cognitive and motor function of patients with mild to moderate PD and is better than the stretching group.

Research has shown that practising Wuqinxi can effectively improve the executive ability of middle-aged and older people [24]. This is similar to our research. The Stroop test in this study is a measure of executive function, which assesses attention control and processing speed (reaction time) during task interference (colour and word inconsistency) [25]. Impaired performance on Stroop is related to a higher risk of subsequent dementia in nonpsychotic patients [26, 27]. Therefore, 12-week Wuqinxi intervention can improve Stroop scores, indicating that Wuqinxi exercise may reduce the risk of dementia in PD patients. Liang [28] evaluated the cognitive function of middle-aged and older people by exercising four times a week for 30 minutes and found that Wuqinxi has a good effect on the cognitive function of middle-aged and older people. In addition, during the 6-month Wuqinxi exercise, the Wuqinxi group's MMSE, visual space and executive ability, naming, attention, delayed recall, orientation, and MOCA score all changed significantly [29].

Cognitive test results of MOCA and FAB show the positive effect of Wuqinxi can be attributed to its essence. As a mind-body aerobic exercise, compared with traditional exercise methods (such as resistance training, muscle endurance training, and strength training), PD patients are required to control their breathing in addition to mobilizing external muscle activities during the exercise; when the practitioner reaches a relaxed state, it can change the mental state of PD patients during practice and improve their bioelectric current and body activity so that the brain waves in various areas of the brain tend to be synchronized and the electromagnetic activity of brain cells is highly effective [30, 31]. The decline in cognitive function in patients with Parkinson's disease represents functional connectivity in the cortex-striatal network accompanied by degeneration of the nervous system other than the dopaminergic system, such as the cholinergic and noradrenaline pathways [32]. Therefore, by instructing patients to focus on their sensory feedback during Wuqinxi exercises, the function of the frontal regions involved in attention and emotional processing may be notably improved.

Another important clinical finding is that current randomized controlled trials have improved the severity of motor symptoms after exercise regimens. Specifically, from the pretest to the posttest, the UPDRS-III score of patients with ON state in Wuqinxi group dropped by 33%. As a bionic traditional movement, Wuqinxi needs to imitate the ferocity of tigers, the briskness of deer, the vigorousness of bears, the agility of birds, and the agility of apes [33]. The movement involves both upper and lower limbs and also emphasizes symmetrical posture, which can strengthen the coordinated contraction of active muscles and antagonist muscles and play a role in the recovery of patients' motor function [34]. A meta-analysis showed that Wuqinxi can reduce antagonistic muscle contraction and improve sport function [11]. After 8 weeks of intensive intervention in 16 patients with Parkinson's disease, Lee et al. found a significant improvement in UPDRS-III in the healthy qigong group compared with the stretching group, which is consistent with the results of this study. [35]

The teaching process is divided into the following: the first three weeks for the learning phase of the movement, 4–6 weeks for the consolidation phase of the campaign and gradually integrating the concepts of breathing and imagination, and 7 to 12 weeks for the collective exercise time of the change. Simple and easy-to-understand actions make it easier to increase patient participation. The gradual process enabled the patients to show good enthusiasm in the process of involvement, and there was some excellent feedback after the course.

### 4.1. Study Limitations and Perspectives

This study has certain limitations. First of all, due to the particularity of the disease, the Wuqinxi group and the stretching group have a small sample size, which may affect the results. Secondly, we did not follow up the patients and could not obtain the durability of the treatment effect. In the future, we can consider adding some brain function imaging technologies, such as near-infrared, EEG, and other advanced instruments, to further explore the impact of Wuqinxi exercise on specific brain areas of patients.

## 5. Conclusions

These preliminary data indicate that the Wuqinxi exercise for 90 minutes twice a week for 12 weeks is feasible, widely accepted, and useful for patients with mild to moderate Parkinson's disease. In the future, further large-scale and controlled studies will be needed to confirm these data to further explore the mechanism of the Wuqinxi exercise on PD improvement.

## Figures and Tables

**Table 1:** Mean (SD) for baseline characteristics of participating patients.

	Wuqinxi	Stretching	*P* value
Number	15	15	
Age (yr)	68.67 ± 4.33	66.93 ± 3.36	0.23
Gender (M/F)	8/7	12/3	
Disease duration (yr)	6.27 ± 4.49	7 ± 3.92	0.63
H&Y scale	1.86 ± 0.83	2.2 ± 0.70	0.24
Education level	10.53 ± 1.99	10.20 ± 1.69	0.84
Antiparkinsonian medications taken (no.)			
Levodopa or carbidopa	14	14	
Pramipexole	7	10	
Others	6	4	
There is no statistical difference in any characteristic category between the two groups			

H&Y, Hoehn and Yahr.

## Data Availability

The data used to support the findings of this study are available from the corresponding author upon request.
